# Evaluation of the Trend of Deformation around the Kanto Region Estimated Using the Time Series of PALSAR-2 Data †

**DOI:** 10.3390/s20020339

**Published:** 2020-01-07

**Authors:** Takashi Nonaka, Tomohito Asaka, Keishi Iwashita, Fumitaka Ogushi

**Affiliations:** 1 College of Industrial Technology, Nihon University, Chiba 2758575, Japan; asaka.tomohito@nihon-u.ac.jp (T.A.); iwashita.keishi@nihon-u.ac.jp (K.I.); 2 Harris Geospatial Co., Tokyo 113-0033, Japan; fogushi@harris.com

**Keywords:** synthetic aperture radar, time-series analysis, small baseline subset, PALSAR-2, displacement

## Abstract

In the Kanto region of Japan, a large quantity of natural gas is dissolved in brine. The large-scale production of gas and iodine in the region has caused large-scale land subsidence in the past. Therefore, continuous and accurate monitoring for subsidence using satellite remote sensing is essential to prevent extreme subsidence and ensure the safety of residences. This study focused on the small baseline subset (SBAS) method to assess ground deformation trends around the Kanto region. Data for the SBAS method was acquired by the Advanced Land Observing Satellite (ALOS)-2 Phased Array type L-band Synthetic Aperture Radar (PALSAR)-2 from 2015 to 2019. A comparison of our results with reference levelling data shows that the SBAS method underestimates displacement. We corrected our results using linear regression and determined the maximum displacement around the Kujyukuri area to be approximately 20 mm/year; the mean displacement rate for 2015–2019 was −7.9 ± 2.9 mm/year. These values exceed those obtained using past PALSAR observations owing to the horizontal displacement after the Great East Japan Earthquake of 2011. Moreover, fewer points were acquired, and the root mean-squared error of each time-series displacement value was larger in our results. Further analysis is needed to address these bias errors.

## 1. Introduction

The south Kanto gas field contains a large quantity of natural gas in the water, which distributed widely across the Kanto region of Japan [1]. The natural gas produced in the South Kanto gas field accounts for more than 90% of the total Japanese production of natural gas dissolved in water. Since the 1930s, gas-production activities have been undertaken by private companies [2]. Large-scale production of gas and iodine began in the mid-1950s in Mobara-shi (the Kujyukuri area) of the Kanto region. This production involved the withdrawal of groundwater from depths of 500 to 2400 m [1]. Consequently, large-scale land subsidence began to occur in the 1960s, and serious land-subsidence surveys were started in the late 1960s [3]. From 1970 to 2010, the maximum accumulated subsidence was 1.0 m, and the subsided area spanned 800 km^2^ [3]. It was reported that the trend of land subsidence has reduced in recent years owing to the efforts of administrations and companies [4]. Land subsidence causes the collapse or tilting of buildings, discontinuities in pipelines, and intrusion of seawater into freshwater aquifers. That is, in addition to the destruction of residential and industrial facilities, it also causes environmental problems and geological disasters. Therefore, understanding land deformation trends caused by human action is significant for sustainable development.

One of the methods for monitoring large-scale ongoing deformation effectively at a low cost is remote sensing. In contrast, conventional levelling can provide precise measurements. The use of the differential interferometry synthetic aperture radar (DInSAR) is a well-known technique for measuring surface deformation [5,6]. However, spatial and temporal decorrelation severely restrict the use of 2-, 3-, and 4-pass DInSAR [7]. The small baseline subset (SBAS) technique is an extension of conventional DInSAR methods that reduces the effects of the decorrelation [7,8]. This technique is intended to be used for the analysis of distributed targets. Although the result of SBAS resembles that generated by conventional DInSAR, the key difference is that SBAS enables the analysis of large time series data. Another deformation monitoring method, especially in urban areas, is the persistent scatterers (PS) technique [9,10]. SBAS is less sensitive to the number of acquisitions than PS because it exploits spatially distributed coherence instead of exclusively considering single points. In addition, SBAS is not limited to linear displacement.

A radar sensor operates in the microwave domain. Different satellite SAR systems operate at different frequencies, which range from 3 MHz to 300 GHz. The longer the wavelength, the greater the ability to penetrate the materials. In previous studies, the SBAS technique has been applied to Phased Array type L-band Synthetic Aperture Radar (PALSAR) data to evaluate the surface deformation around the Chiba Prefecture [11,12]. For example, 26 PALSAR scenes were utilized to monitor deformation around the Kujyukuri area from 2006 to 2010 [11]. According to the analysis of the data, a displacement of more than 15 mm/year was observed around the cities of Yachimata and Oamishirasato. These displacement values were compared with GNSS Earth Observation Network System (GEONET) data at 12 validation points; the results showed that the root mean square error (RMSE) was approximately 10 mm. 

PALSAR-2 on the Advanced Land Observing Satellite (ALOS)-2 has been operated since 2014 and is expected to be used for many applications, especially disaster management [13,14]. The orbit can be controlled with high accuracy, enabling improved coherence in PALSAR-2 in comparison with PALSAR [15]. In previous studies, displacement was evaluated using DInSAR, and large displacement errors occurred depending on the pairs of images [16]. However, knowledge regarding the applicability of the SBAS technique and the errors in the mean displacement velocity, as well as the difference between the results acquired from PALSAR-2 and PALSAR, were not gathered fully in previous studies. Therefore, the purpose of this study was to evaluate the displacement accuracy using PALSAR-2 data and reveal the displacement in the Kujyukuri area by this method. Additionally, we discussed the applicability of the SBAS technique to the PALSAR-2 data by comparing the acquired results with those of PALSAR.

## 2. Study Area and Used Data

### 2.1. Study Area

**Figure 1:**
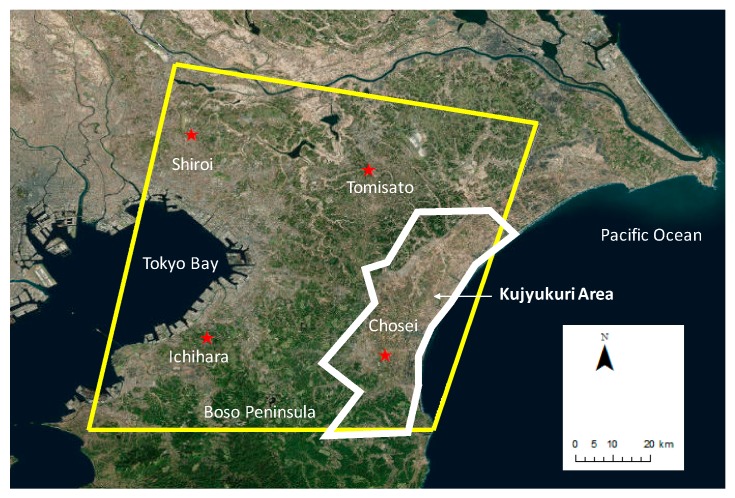
Location of the study site (yellow box). The red stars indicate GeoNET stations.

Figure 1 shows the study area (demarcated by a yellow box in the figure), which encompasses most of the South Kanto gas field. It includes a large part of the Chiba Prefecture and small parts of Tokyo, Kanagawa, Saitama, and Ibaraki. A large part of the prefecture is made up of the Boso Peninsula, which shields Tokyo Bay from the Pacific Ocean. The northern and western parts of the Boso Peninsula are highly urbanized, which has caused overpopulation in this area (the total population of the Chiba Prefecture was more than 6.2 million in October 2018 [17]). The elevation of this area ranges only from 0 m to approximately 300 m, and the topography is steep. Even today, most of the natural-gas development in Chiba is concentrated in the Kujyukuri area, which is on the east coast of the Boso peninsula.

### 2.2. Used Data

The ALOS-2, developed by Japan Aerospace Exploration Agency (JAXA), was launched on 24 May 2014 as the successor to ALOS-1 (launched on 24 January 2006 and decommissioned in 12 May 2011). ALOS-2 was designed for many applications, such as disaster monitoring, land monitoring, agricultural monitoring, natural resource exploitation, and global forest monitoring. ALOS-2 is equipped with PALSAR-2, which provides imaging with a high spatial resolution of 1–3 m via the spotlight and Stripmap mode. ALOS-2 has a 14 day revisit cycle, which makes it three times faster than ALOS (with a 46 day cycle). Furthermore, the high-accuracy orbit control, which is maintained within a 500 m radius tube for the reference orbit, enables high coherence for InSAR processing [16]. Table 1 lists the acquisition parameters used in this study. We collected 15 PALSAR-2 scenes from 15 January 2015 to 7 March 2019 (Table 2) for the InSAR stacking process. All the scenes were observed in the ultrafine single mode (with a 3 m spatial resolution), the descending direction, and right-looking mode.

**Figure 2:**
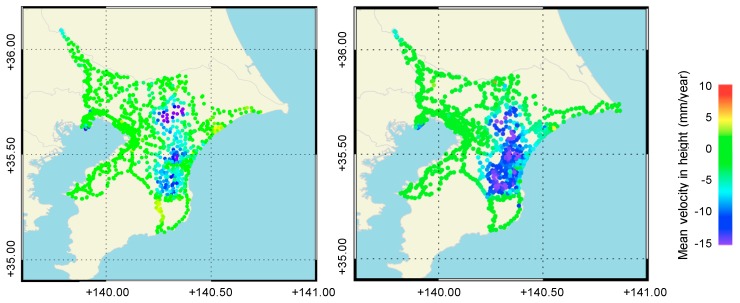
Mean subsidence velocity acquired from the levelling data for Chiba Prefecture from 2015 to 2019 (**left**) and from 2006 to 2010 (**right**).

The Geospatial Information Authority of Japan (GSI) has been operating a network of continuous Global Navigation Satellite Systems (GNSS) stations called GEONET [18]. We exploited GEONET and the levelling survey measurement data [19] to validate the InSAR derived deformation. Four points (93018: Shiroi, 93020: Tomisato, 93030: Ichihara, and 93033: Chosei) were selected for validation from the daily GPS data provided by the GSI (Figure 1). Levelling survey measurements were conducted in the Chiba Prefecture annually to understand the status of land deformation in the Chiba Prefecture. Figure 2 shows the mean velocity map derived from the levelling data for the study site from 2015 to 2018 and from 2006 to 2010. It can be seen that the spatial trend almost agrees between these durations, although the mean velocity decreased for the former period. Furthermore, subsidences of more than 10 mm/year appear to have occurred at several points around the Kujyukuri area [20].

## 3. Method

**Figure 3:**
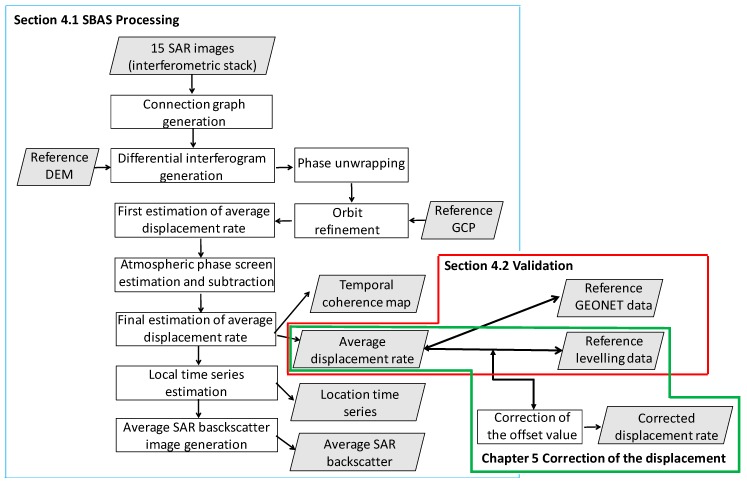
Flowchart of the short baseline subset method used in this study. DEM, digital elevation model; GCP, ground control point.

Figure 3 shows the flow of the study. We used the SARScape module of the ENVI 5.5 software, provided by Harris Geospatial Solutions, to perform the SBAS analysis. The SBAS workflow included the following seven steps [21,22]: the creation of a connection graph (generating all differential interferograms from the input image stack that fulfil the criteria for temporal and geometric baselines within a given interval in time or for a normal baseline with respect to the critical one), differential interferogram generation (spectral shift and adaptive filtering), phase unwrapping (either with a conventional 2D approach or a combined 3D approach), orbit refinement and reflattening, first estimation of the average displacement, atmospheric phase screen, and final estimation of the average displacement. In this study, we achieved a ground resolution of 8 m by using a multi-looking factor of three in the range and four in the azimuth. The topographic phase was removed using a 30 m digital elevation model (DEM) developed by the Shuttle Radar Topography Mission (SRTM) [23], which has been used for processing previous PALSAR data, as well. We applied the Goldstein filter [24] to smooth the differential phase and applied the minimum cost flow (MCF) network [25] along with the Delaunay method to unwrap the differential interferograms. In this stage, the unwrapping coherence threshold was 0.30. SBAS estimated displacement relative to the ground control points (GCPs) used during phase-displacement conversion. A previous study stated that the selection of the reference points is not real issue [7]; we selected approximately one hundred GCPs evenly across the entire image; these GCPs were also used for the PALSAR SBAS processing, refinement, and re-flattening. Subsequently, the average displacement rate and residual height-correction factors were estimated by inverting a linear system, which included all measures (one for each interferogram) together with their proportionality coefficients, depending on the temporal and geometric baseline of each pair [8]. Then, low-pass and high-pass spatial filters were used for the time-series images, to screen and remove the atmospheric phase components. All of the final displacement measurements were conducted in the satellite line of sight (LOS) direction and were geocoded in the WGS84 reference ellipsoid. To validate the displacement rates, we used the GEONET (Section 4.2.1) and levelling (Section 4.2.2) data as references. We established the approximate equation to correct the values based on the relationships between SBAS and levelling. Finally, the corrected displacement value was acquired for each point (Section 5). 

**Figure 4:**
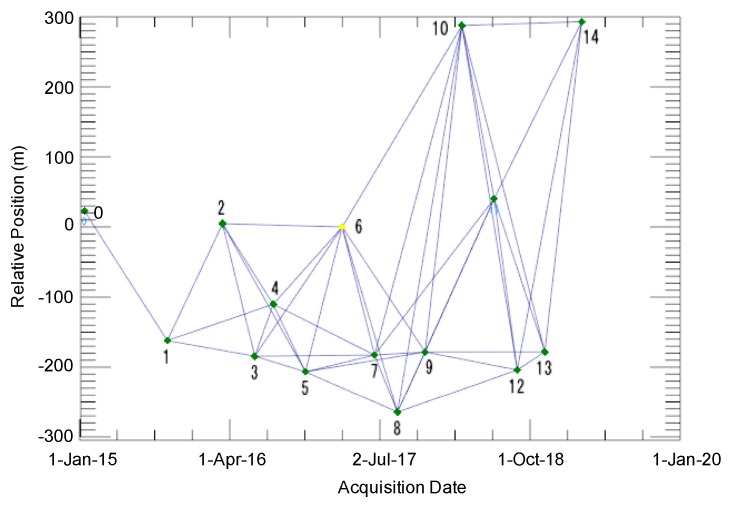
Temporal and spatial baseline distributions of the SAR interferogram, acquired from 15 ALOS-2 PALSAR-2 data points (the normal baseline restriction used was up to 50% of the critical baseline, and the time interval between the interferometric pair was up to 365 days). Each number shows the acquisition date (Table 2).

Figure 4 shows the temporal and spatial baseline distributions of the SAR interferogram from the ALOS-2 PALSAR-2 dataset, in which each acquisition is represented by a diamond associated with an ID number; the green diamonds represent valid acquisitions, while the yellow diamonds represent the super master image (this image is the reference image for the entire process, and all the processed slant range pairs are co-registered on this reference geometry). Using the PALSAR-2 data, 45 interferograms were generated for the SBAS processing with respect to the multi-master images. The threshold criterion for obtaining the absolute maximum normal baselines was half that of the critical baseline (≒19.3 km), and that for the absolute maximum temporal baselines was 365 days. The image acquired on 9 March 2017, was chosen as the super master image. The maximum baseline among all the pairs was approximately 500 m, which satisfied the threshold criterion of the spatial baseline. 

## 4. Results

### 4.1. Displacement Rates

**Figure 5:**
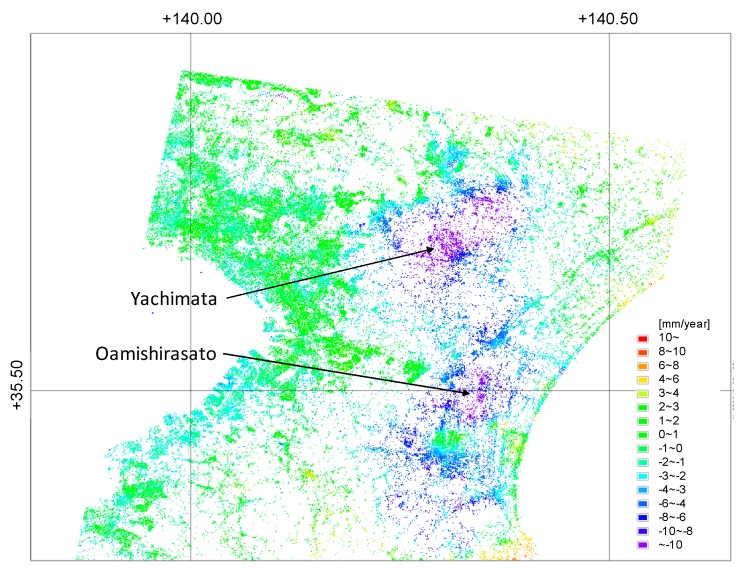
Mean displacement velocity from 2015 to 2019, derived from the PALSAR-2 data [26].

Figure 5 shows the estimated mean displacement velocity from 2015 to 2019. Positive values indicate movement toward the satellite. Displacement was rarely observed around the coast of Tokyo Bay, while obvious displacement was observed around the Kujyukuri area. The mean displacement rate in the study area was −1.4 ± 3.2 mm/year. The results show that 75% of the points indicate displacement rates between −3 mm/year to 3 mm/year [26]. From 2006 to 2010, the displacement rates for this area derived from PALSAR data were −3.3 ± 5.8 mm/year in previous studies [11]. 

Some studies have obtained displacement values even for vegetated areas using longer L-band wavelengths [27,28]. However, using PALSAR-2, vegetated areas could not be observed due to lower coherence. Further, PALSAR-2 operates by acquiring data on fewer points than that acquired by PALSAR. A reason for this could be the larger RMSE of each time-series displacement value in the PALSAR-2 dataset (Section 4.2). Further analysis is required to understand the pertinent conditions, such as the number of images, threshold coherence, and the threshold for the spatial and temporal baseline. 

### 4.2. Validation

#### 4.2.1. Comparison with GEONET Data

**Figure 6:**
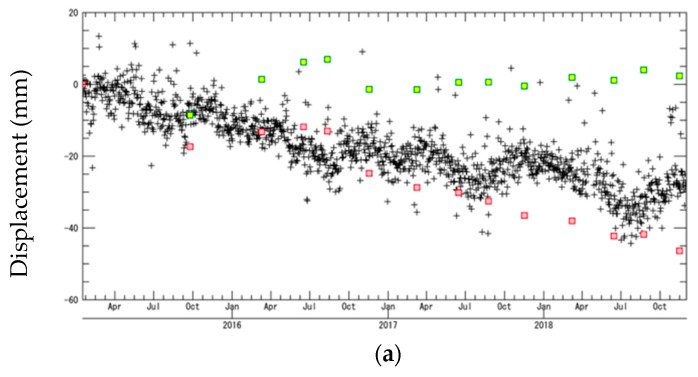

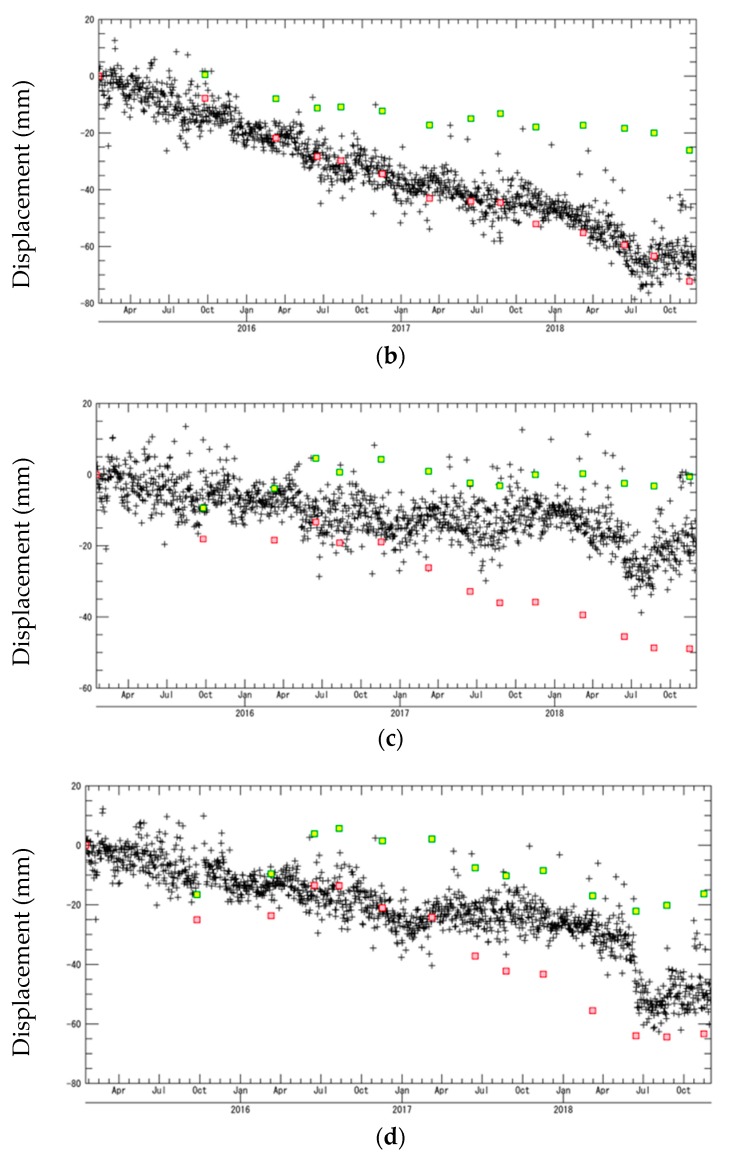
Comparison between the displacement calculated by the small baseline subset (SBAS) method and that obtained from the GEONET data for (**a**) Shiroi, (**b**) Tomisato, (**c**) Ichihara, and (**d**) Chosei. The green and red squares and black crosses represent the SBAS, corrected SBAS, and GEONET data, respectively.

We conducted a quantitative comparison of the time-series displacement values with the GEONET data for four validation points (Figure 1) to assess the accuracy of the InSAR-derived results. We did not use the values of several GEONET points inside the study area because negligible coherence was observed. The values derived from the GEONET data points were converted into values in the line of sight (LOS) direction for comparison (Figure 6). The overall displacement for these four stations indicates that the SBAS method (shown by green squares) underestimated the overall displacement. The displacement in Tomisato and Chosei was continuous, and the total displacement was approximately 50 mm from 2015 to 2019.

The estimated RMSE of the displacement value, which was 20 mm from 2015 to 2019, is twice that of the displacement from 2007 to 2010, and was derived from PALSAR data. Several factors caused errors in the displacement values: atmospheric disturbance [29], interferogram noise [30], and geometric errors in the sensor (specification: 5 m [31]). In the case of InSAR, the interferogram noise (*α*) is estimated by the ratio of DEM error (*DEM _ERR_*) to the height of ambiguity (*dh*/*dϕ*), which is defined by Equations (1) and (2):

(1) $$DEM_{ERR}=\alpha \frac{dh}{d\phi }+\beta ,$$

(2) $$\frac{dh}{d\phi }=\frac{\lambda Rsin\theta }{2Bp}$$

where *λ* is the wavelength, *R* is the slant-range length, *θ* is the angle of incidence, and *Bp* is the perpendicular baseline. Previous studies have revealed that the interferometric phase noise of PALSAR-2 is 2.0%, half that of PALSAR [15,32,33]. The phase noise displacement error was estimated to be 3 mm by using a noise ratio of 2.0% in the case of PALSAR-2, which is much smaller than the RMSE displacement error of 20 mm. The geometric errors were estimated using a spatial deviation of 3 × 3 pixels around the validation points and were found to be less than 0.6 mm.

#### 4.2.2. Comparison with Levelling Data

**Figure 7:**
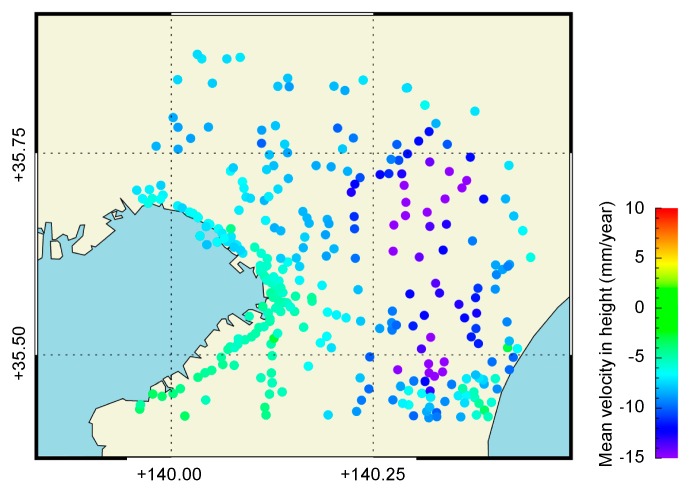
The reference mean velocities in the land observing satellite (LOS) direction calculated by the levelling data and GEONET data.

**Figure 8:**
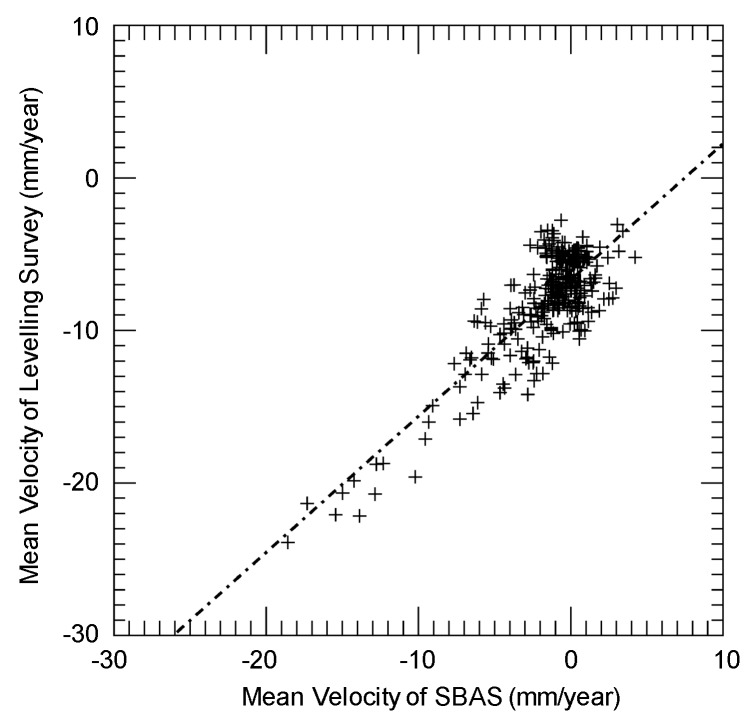
Comparison between the mean velocities obtained using the SBAS method and the levelling data.

The mean velocity was quantitatively compared with the levelling data for 310 points. First, we took the leveling data for the same periods of study and converted the data into values in the LOS direction using the horizontal displacement from the nearest GEONET data (Figure 7). It should be noted that the reference levelling data were temporally too sparse (one data per year) to enable acquisition of the mean velocity. Figure 8 shows the comparison between SBAS-derived linear subsidence rates and the levelling data. The results show that the SBAS method underestimated the velocity for the whole range from −25 to 0 mm/year, and the RMSE is 6.8 mm/year. This trend can be expressed by the following linear regression, and the correlation coefficient is 0.82:

(3) $$V_{LEVEL}=0.89V_{SBAS}-6.69$$

where *V_LEVEL_* is the mean velocity per level, and *V_SBAS_* is the velocity calculated by SBAS. 

## 5. Discussion

**Figure 9:**
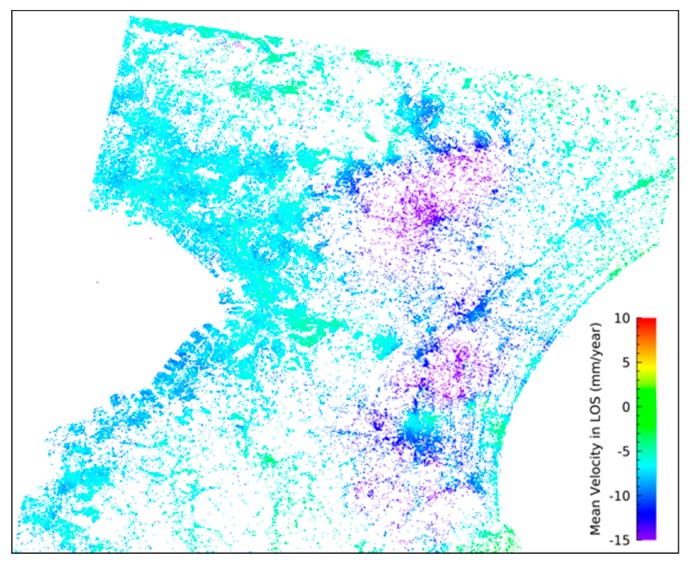
Corrected mean displacement velocity from 2015 to 2019, derived from the PALSAR-2 data.

In this article, we present a method for correcting the offset in SBAS-derived mean displacement velocity values using levelling data. According to the analysis presented in the previous section, we acquired the relationships (Equation (3)) between the SBAS and levelling data. Using Equation (3), we calculated the corrected mean displacement velocity values, as shown in Figure 9. The average and standard deviation of the corrected mean velocities were −7.9 mm/year and 2.9 mm/year, respectively. The spatial distribution of the mean velocity agreed with that of the levelling data (Figure 7). This implies that the SBAS-derived mean displacement velocity can supplement the levelling data subsequent to correction, even when the number of images is not sufficient. The time evolutions of SBAS were also corrected using the coefficient values derived in Equation (3). The amount of displacement can be obtained by calculating the difference in the mean velocity between the corrected and original SBAS values. The difference in the mean velocity can then be converted to offset the displacement by multiplying time, and the corrected SBAS displacement, *Disp_CORRECTED SBAS_*, can be calculated, as described in Equation (4):

(4) $$Disp_{CORRECTED\;SBAS}=Disp_{SBAS}+\left\{\left(0.89V_{SBAS}-6.69\right)-V_{SBAS}\right\}T$$

where *Disp_SBAS_* is the original SBAS displacement, *V_SBAS_* is the mean velocity of the original SBAS displacement, and *T* is the length of time from the start of the observation. The corrected SBAS displacements are shown in red in Figure 6, and the time evolutions were better fitted to the GEONET data points than the original SBAS displacements in Figure 6a,b,d. However, the corrected SBAS displacement in Figure 6c was more overestimated than the GEONET displacement. According to the GEONET displacements in Figure 6, there are some types of displacements (linear and non-linear) in this study area, and these types are locally changed within a scene. This could be a potential reason why the estimated displacements by SBAS have some offsets with respect to the ground truth data.

Table 3 displays the differences between the validated mean displacement velocities of the values obtained from the corrected PALSAR-2, the original PALSAR-2, and the PALSAR data points. The RMSE of the PALSAR-2 data was twice that of the PALSAR data because of the bias in the displacement derived from the PALSAR-2 data. However, the high correlation coefficient for the linear regressions indicates that the deviation from the regression is relatively small. Therefore, after correcting the mean displacement velocity using linear regression, the RMSE of the PALSAR-2 data was reduced to 2.0 mm/year, almost half that of the PALSAR data. Based on this result, we assumed that the temporal displacement errors of PALSAR-2 were also reduced to half those of PALSAR. If this assumption is correct, our results are in agreement with the results of a previous study in which the phase noise of PALSAR-2 was found to be half that of PALSAR [15]. These results indicate the feasibility of using both PALSAR-2 and PALSAR data for displacement estimation using the SBAS technique. However, the accuracy of the obtained results is not good compared to those obtained from previous studies conducted on C-band satellites due to the following reasons: (1) The sensitivity of the L-band interferograms to surface deformation is much lower than that of the C-band [7]. (2) The observation frequency of PALSAR-2 is low (approximately four observations per year) compared with other C-band satellites, such as ERS-1, ERS-2, and Envisat [34,35].

**Figure 10:**
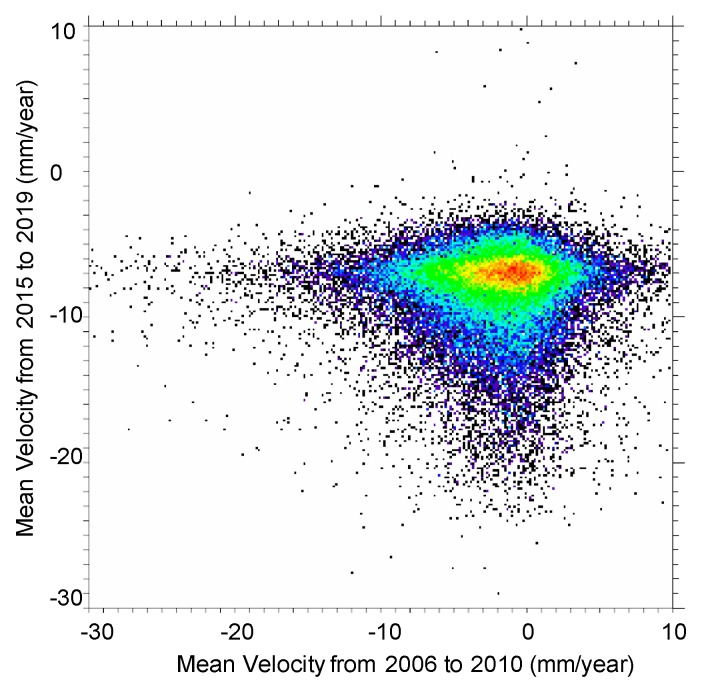
Comparison between the mean displacement velocity from 2015 to 2019, derived from PALSAR-2 data, and from 2006 to 2010, derived from PALSAR data.

Finally, it is worth determining whether the deformation patterns of PALSAR-2 and PALSAR are consistent with each other, although the acquisition period does not overlap. Therefore, the displacement values during the two time periods were compared. The acquisition conditions for the PALSAR data were very similar to those of the PALSAR-2 data: a 34.3° incidence angle and descending path. Figure 10 shows a comparison between the deformation rates in the LOS direction obtained from the PALSAR-2 and PALSAR data. A positive correlation can clearly be seen between them. Most of the subsidence values ranged from −5 to 5 mm/year from 2006 to 2010 and shifted to −10 to −5 mm/year from 2015 to 2019. The reason for the increase in the deformation rates despite a decrease in the subsidence measured by levelling during this period (Figure 2) is that the horizontal displacement increased after the extensive Great East Japan Earthquake in 2011. 

## 6. Conclusions

To monitor the temporal changes in land subsidence caused by the extraction of natural gas, this study estimated the land displacement within the Chiba Prefecture from 2015 to 2019 using time-series satellite SAR data. We adopted the SBAS technique, and fifteen PALSAR-2 data points were used. The estimated displacement had a similar spatial trend to that derived from PALSAR. In contrast to previous studies using PALSAR data, the number of observed points in the present study was low. This can be explained by the observation that a substantial number of points of PALSAR-2 depicted a non-linear trend for each time series displacement value. This, in turn, was attributed to the insufficient number of images. This study quantitatively evaluated the accuracy of the annual displacement rate determined using PALSAR-2 data by comparing this data with in-situ levelling survey data. The estimation accuracy was found to be 6.8 mm/year, and PALSAR-2 underestimated the displacement values. After correction of the bias via linear regression, the RMSE of the displacement velocity derived using the SBAS method was reduced to 2.0 mm/year, approximately half that of PALSAR. The maximum velocity of displacement derived from the map subsequent to the bias correction was found to be approximately 20 mm/year in the Kujyukuri area for the period 2015–2019; however, this value is higher than that obtained for the 2006–2010 period. This can be explained by an increase in the horizontal displacement after the Great East Japan Earthquake that occurred in 2011. Through this study, we confirmed the applicability of the SBAS-derived displacement rates to PALSAR-2, as well as their reliability despite an insufficient number of PALSAR-2 images. In addition, this study quantitatively revealed the displacement errors using an extensive network of levelling data. Additional acquisition results in better product quality because it allows for better estimation and a reduction of the atmospheric component. However, to determine the causes of the bias errors obtained, further analyses are required. We intend to continue monitoring the temporal changes in displacement in the study area through SBAS analysis using PALSAR-2 data in future studies.

## Figures and Tables

**Table 1:** Acquisition parameters of the Advanced Land Observing Satellite (ALOS-2) Phased Array Table 2. dataset.

Band (wavelength)	L-band (24 cm)
Operation mode	Ultrafine
Polarization	HH
Resolution	3 m
Swathe	50 km
Off-nadir angle	32.8°
Orbit direction	Descending
Revisit cycle	14 days

**Table 2:** List of used PALSAR-2 data (path number: 18).

No	Acquisition Date
1	15 January 2015
2	24 September 2015
3	10 March 2016
4	16 June 2016
5	11 August 2016
6	17 November 2016
7	9 March 2017
8	15 June 2017
9	24 August 2017
10	16 November 2017
11	8 March 2018
12	14 June 2018
13	23 August 2018
14	15 November 2018
15	7 March 2019

**Table 3:** Comparison between the validated mean displacement velocities of the values obtained Figure 2. original PALSAR-2, and PALSAR data.

	Corrected PALSAR-2	Original PALSAR-2	PALSAR
Time	2015–2019		2006–2010
Number of points	310		519
RMSE of the mean velocity	2.0 mm/year	6.8 mm/year	3.5 mm/year
Linear regression slope	1.00	0.89	0.96
Linear regression intercept	−0.03	−6.69	−1.44
Correlation coefficients	0.82		0.70
